# Spatial tick bite exposure and associated risk factors in Scandinavia

**DOI:** 10.1080/20008686.2020.1764693

**Published:** 2020-06-07

**Authors:** Solveig Jore, Sophie O. Vanwambeke, Daniel Slunge, Anders Boman, Karen A. Krogfelt, Martin Tugwell Jepsen, Line Vold

**Affiliations:** a Department of Infection Prevention & Preparedness, Norwegian Public Health Institute, Oslo, Norway; b Georges Lemaître Centre for Earth and Climate Research, Earth & Life Institute, Louvain-la-Neuve, Belgium; c Center for Sustainable Development, GMV, University of Gothenburg, Gothenburg, Sweden; d Department of Economics, University of Gothenburg, Gothenburg, Sweden; e Department of Virus and Microbiological Diagnostics, Statens Serum Institut, Copenhagen, Denmark; f Dept of Science and Environment, Roskilde University, Roskilde, Denmark

**Keywords:** *Ixodes ricinus*, tick, tick bite, Norway, Sweden, Denmark

## Abstract

Tick-borne diseases are emerging and re-emerging threats causing public health concerns in Europe and North America. Prevention and control requires understanding of human exposure and behaviour. The aim was to measure exposure to tick bites across Scandinavia, its spatial distribution and the associated risk factors.

Methods

We sent a web-based survey to a randomly chosen population and analysed answers by Principal Component Analysis and Chi-Square. Individual responses were aggregated at the municipality level to assess the spatial distribution of bites.

Results

Nearly 60% of adults reported bites at low levels (1-5 bites); however, the majority were not in their resident municipality. We found two spatial profiles: In their home municipalities, people were most often bitten in less, but not the least, urbanized areas. When visiting other municipalities, people were most frequently bitten in peri-urban areas. Running/walking in the forest, gardening, and paddling/rowing were activities most strongly associated with bites.

Conclusion

Tick bites affect the entire Scandinavian population, with a higher risk in Sweden compared to Denmark and Norway. The frequency of observation of ticks in the environment or on pets might be used as a proxy for the actual risk of exposure to tick bites. Our results indicates that urban-dwelling outdoor enthusiasts and inhabitants of rural areas must be equally targeted for prevention campaigns.

## Introduction

Ticks are the second most important vectors of pathogens in both humans and animals that transmit an extensive range of viral, bacterial, and protozoan pathogens. They are, along with the pathogens transmitted, a major threat to animal and human health [1,2]. Ticks are currently expanding their distribution in Scandinavia [3,4]. The emergence of new tick-borne diseases and the re-emergence of existing ones are now public health concerns in Europe and North America [1,5]. In Scandinavia as elsewhere in Europe, the tick *Ixodes ricinus* is the main disease vector in humans and Lyme borreliosis (LB), caused by the spirochete bacteria of the *Borrelia burgdorferi sensu* lato (s.l) complex, the most common tick-borne disease in Europe.

Associations between tick bites and behavioural and environmental risk factors are challenging to assess, particularly with regard to intensity of human exposure. In addition, the relationship between tick bites and risk of tick-borne diseases is poorly known, since only a fraction of bites lead to disease and those who develop disease are not all diagnosed. Hence, information on human encounter rates with infected ticks is almost impossible to obtain. Identifying areas with high numbers of tick bites along with predisposing behaviours or activity, together with reported incidence of diseases, can provide important information on the possible risk of acquiring tick-borne disease and the at-risk population in order to target communications on preventive behaviour.

There is a lack of published results on the occurrence of tick bites and their determinants on regional level in Europe. Existing published data in Scandinavia, although poor spatially and temporally, indicate that tick bites are a widespread phenomenon. No study on tick bite exposure has been published so far from Denmark and there is only one minor study from a single county in Norway, whilst there exist a few studies from Sweden, these were however published years ago. The risk of human exposure to tick bites might also have changed in the recent years due to the ongoing global environmental change.

In a telephone-based survey one in five (18%) Swedish residents experienced one or more tick bites during the tick season in 2005 [6]. The incidence and temporal pattern of tick bites were further surveyed in a population in south-eastern Sweden (during 2000–2001). This study found a 4% risk of being bitten per 10 hours spent outdoors [7]. Later (2008–2009), a study among 34 primary health care centres in Sweden and Åland Islands recorded tick bites in 55% of respondents [8]. Of these, 68% had between one and four bites and 13% had more than 10 bites. A greater proportion of participants from Åland Islands (18%) reported over 10 tick bites compared to southernmost Sweden (10%) and south to central Sweden (8%). Further, in a nation-wide survey from 2013, 68% of respondents reported tick bites [9], with only 25% of respondents living in northern Sweden reporting a tick bite, compared to around 74% in southern Sweden. In the same study, 11% of the respondents reported ever being diagnosed with LB and 1% had been diagnosed with TBE or other tick-borne diseases [9]. In Norway, 66% of blood donors in one western county had experienced tick bites during their life time [10]. In the Netherlands retrospective cross-sectional studies in 1994, 2001, and 2005 determined the geographical distribution of tick bites consultations and incidence of erythema migrans (early symptom of LB) among the general population. They estimated that every year people in the Netherlands suffer approximately 1–1.3 million tick bites [11].

In this study we aimed at producing region-wide measures of exposure to tick bites using a large survey that sampled representative populations of Denmark, Norway, and Sweden. The second aim was to study the spatial distribution and associations between tick bites and outdoor activities, demographic characteristics, and protective measures.

## Materials and methods

### Study design and data collection

As part of a larger Scandinavian research project on ticks and tick-borne diseases (ScandTick Innovation; http://scandtick.com/), a comprehensive web-based survey (https://snd.gu.se/en/catalogue/study/snd1119) was designed to study exposure to and experience of ticks and tick bites, protective behaviour and risk perceptions among people in Denmark, Norway, and Sweden. The survey included 48 questions [12] in the following categories: exposure to ticks; having had a tick-borne disease; knowledge on tick-related issues, general trust and risk preferences; protective behaviour related to tick bites; recreational behaviour; and demographic characteristics (age, gender, education, income, and country of residence). Respondents were asked to indicate general trust and willingness to take risks on an ordinal scale. Such scales have successfully been used to measure risk perceptions [13]. We focus here on the subset of the questions in the survey relating to tick bite exposure and tick-borne disease. Results regarding protective behaviour and knowledge and risk perceptions related to tick bites, LB, and tick-borne encephalitis have been published in two other papers [12,14].

Survey respondents were randomly selected from national telephone registries within each country ensuring a representative sample regarding age, sex, and regions. The targets for each sub-quota within each country were based on the demography of the respective country, extracted from the national registries (www.ssb.no, www.dst.dk and www.scb.se). We targeted 750 respondents from Norway and Denmark and 1000 respondents from Sweden. The Norwegian Public Health Institute, The Danish Public Health institute, and University of Gothenburg in Sweden hired a private survey company. The company telephoned 5096 people in Denmark, 7194 in Norway and 9901 in Sweden. Respectively (for all the following numbers) 1518 (21%), 1436 (28%), and 2037 (21%) were willing to participate and were sent the electronic survey. 250 (16%), 157 (11%), and 214 (11%) emails bounced back, so 1268, 1279, and 1823 actually received the survey. Of these, 783, 789, and 1096 completed the survey, giving a mean response rate of 61%. The demographics of the final sample is given in tables 1, 2, and 3 and in companion papers [12,14].

**Table 1.** Gender distribution.

Gender	Norway	Denmark	Sweden	Total
Women	406 (51.4%)	420 (53.6%)	577 (52.6.%)	1403 (52.6%)
Men	380 (48.2%)	361 (46.2%)	514 (46.9%)	1255 (47.0%)
Other	0 (0%)	1 (0.1%)	2 (0.2%)	3 (0.1%)
No answer	3 (0.4%)	1 (0.1%)	3 (0.3%)	7 (0.3%)

**Table 2.** Level of education.

Education	Norway	Denmark	Sweden	Total
Not finished primary school	1 (0.1%)	3 (0.4%)	1 (0.1%)	5 (0.2%)
Primary school	19 (2.4%)	57 (7.3%)	73 (6.7%)	149 (5.6%)
Secondary School	212 (26.9%)	114 (14.6%)	339 (30.9%)	665 (24.9%)
University/High School 1-3 years	198 (25.1%)	249 (31.8%)	283 (25.8%)	730 (27.4%)
University/High School more than 3 years	329 (41.7%)	329 (42.0%)	345 (31.5%)	1003 (37.6%)
PhD	20 (2.5%)	10 (1.3%)	34 (3.1%)	64 (2.4%)
Does not want to answer	10 (1.3%)	21 (2.6%)	21 (1.9%)	52 (1.9%)
Total	789	783	1096	2668

**Table 3.** The age of the respondents.

Age group	Norway	Denmark	Sweden	Total
18–29	134 (17%)	85 (11%)	168 (15%)	387 (15%)
30–44	217 (27%)	192 (24%)	297 (27%)	706 (26%)
45–59	205 (26%)	217 (28%)	277 (26%)	699 (26%)
60+	233 (30%)	289 (37%)	354 (32%)	876 (33%)
All	789	783	1096	2668

### Spatial analysis at the municipality level

We aggregated and mapped relevant variables at the municipality level, overlaid on population density. The survey setup (cross-sectional) did not allow to compute incidence rates. We mapped the number of respondents, the number of times a municipality was quoted as a place different from the municipality of residence where a tick bite was acquired, and the mean number of bites acquired in the municipality of residence (hereafter ‘home municipality’) per respondent, with flags indicating municipalities with no respondents and municipalities with one to five respondents. Population density (persons/km^2^) at the municipality level was computed using population figures from the national statistics institute of each country. The spatial and environmental characteristics of municipalities were measured using the Human Footprint Index (HFP) [15], and distance to major cities, as a summary measure of tick habitat and human exposure, and of accessibility for outdoors recreation users, respectively. The HFP combines remotely sensed data of human land use and infrastructure and summarizes human pressures exerted on natural systems (values ranging from 0 to 50). Municipalities of major urban centres were selected based on two criteria: either the city has over 100 k inhabitants, or the municipality had a density higher than 1000 persons/km^2^. Distances were computed between municipalities and their nearest city as a closest edge-to-edge distance. Associations between these and the tick bite indicators mapped were tested using Kendall’s tau [16].

### Individual analyses

We analysed individual-level data using chi^2^ and principal component analysis (PCA). Chi^2^ were used for testing significant relationships between two variables (e.g. tick bite and gender). PCA was used considering the survey set-up (cross-sectional) does not allow to explore causal relationships. It is not possible to identify dependent or independent variables (e.g. assessing the effect of behaviour on tick bite presence/absence). Instead, we opted to explore correlations across the entire dataset using a PCA. We used all questions (variables) except those questions for which over 90% of respondents gave the same answer, resulting in 36 and 37 variables in Denmark and Sweden, and Norway respectively. In order to use all observations, we imputed missing data in the following way: for education level we used the overall average in the sample, at country level. For income, we used the sample average computed by education category. Income data were missing for 279, 190, and 272 respondents in Denmark, Norway and Sweden, whilst education data were missing for 21, 10, and 21 respondents in Denmark, Norway and Sweden, respectively. Variables were treated as continuous variables and standardised (to avoid scale dependence) before running the PCA. We focused on the interpretation of correlations between original variables and resulting components, assessing which variables group on the components bearing over 5% of the total variability. In the analyses, the respondents bitten outside Scandinavia were excluded. We did not exclude respondents with poor tick knowledge (in our questionnaire) since tick identification and knowledge often is poor in the general population [12,17].

## Results

### General description of the survey sample

Overall, 53% of respondents were women and 47% men (Table 1). The level of education and age of respondents in the different countries are shown in Tables 2 and 3.The descriptive statistics of the country subsample regarding gender, age, and educational attainment are described and discussed in [12].

### Spatial analysis

**Figure 1.**
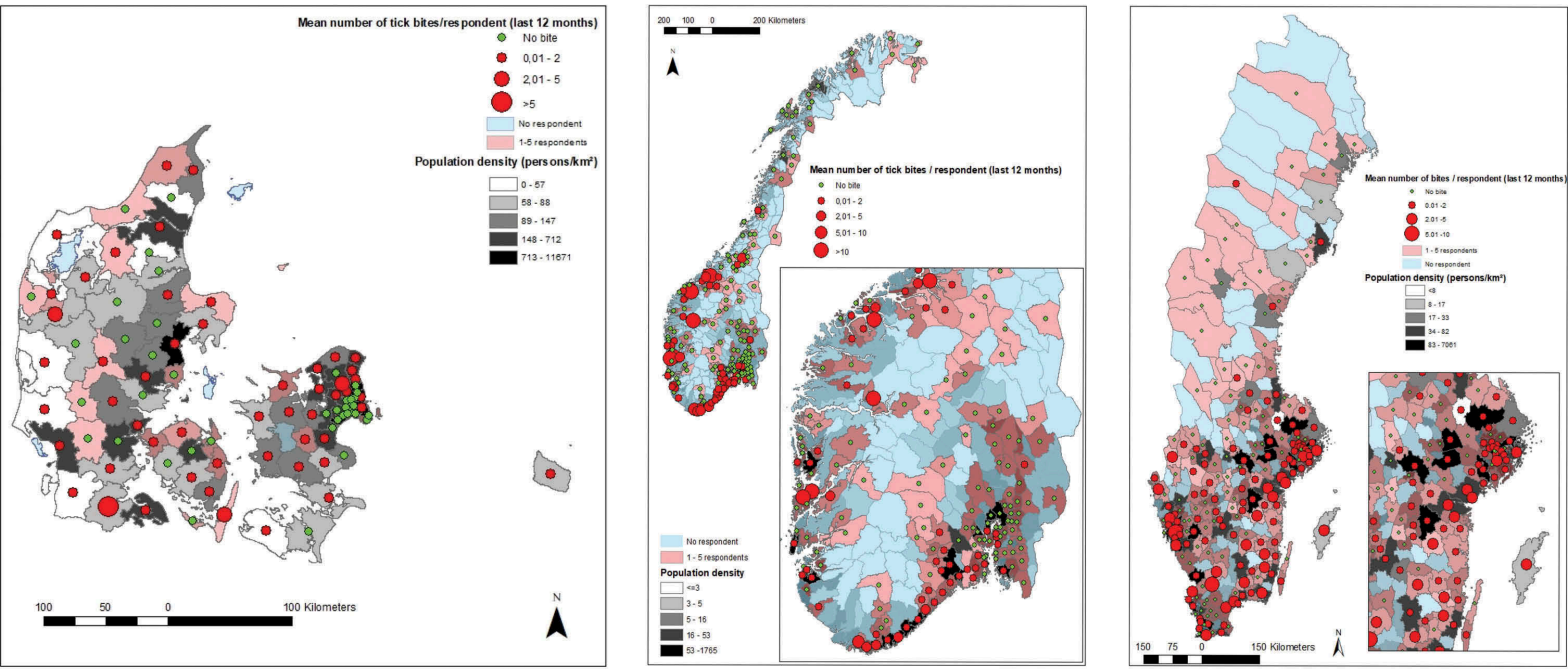
Legend: Mean number of tick bites acquired in the residence (‘home’) municipality displayed over population density (persons/km^2^). Municipalities coloured blue had no respondent in the survey. Municipalities coloured red had one to five respondents. The rest had over five respondents. The figure displays maps for Denmark, Norway and Sweden.

**Figure 2.**
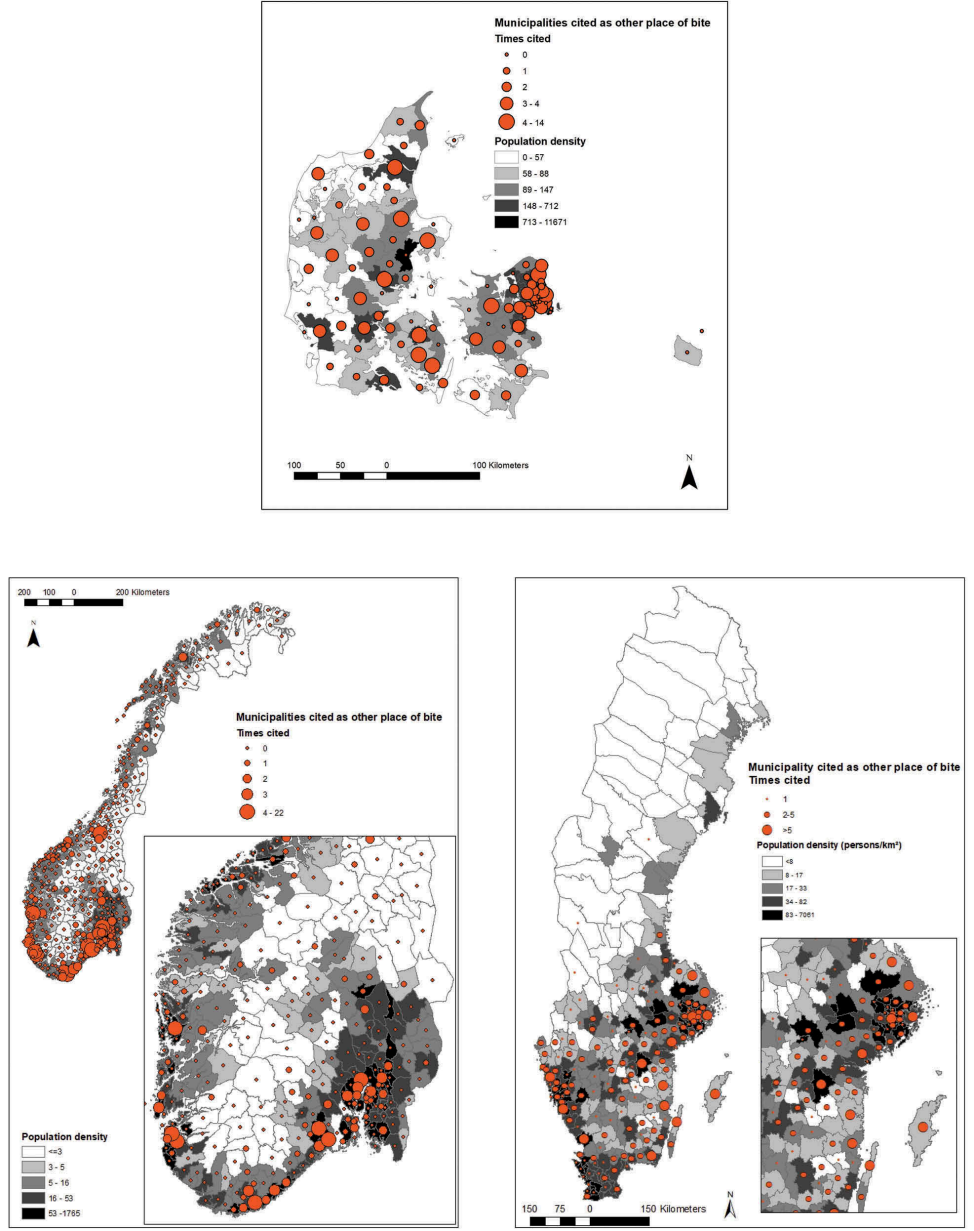
Legend: Number of times a municipality was cited as ‘other place’ of tick bite for respondents not being bitten in the residence municipality displayed over population density (persons/km^2^). The figure displays maps for Denmark, Norway and Sweden.

In Denmark the mean number of bites (during the previous 12 months) acquired in the home municipality varied between zero and six, and was homogeneously distributed across the country. In Sweden, where bites varied between 0 and 10, bites were mostly found in the southern half of the country. Norway had a much larger mean number of bites (0–54.5), with the highest values along the southwestern coast, this was due to a small number of respondents with very high number of bites (Figure 1). In order to evaluate the rural character of municipalities compared to the number of bites reported in the municipality of residence, we plotted them against the mean Human Footprint Pressure (HFP) of the municipality (Supplementary Figure 1). In all countries, the highest mean number of bites at home occurred in municipalities with lower, although not the lowest, mean HFP values. Kendall’s tau between mean HFP and the mean number of bites was significant and positive in Norway and Sweden (Supplementary Table 1). For respondents visiting another municipality (Figure 2), tick bites were more common in municipalities’ closer to major cities, regardless of their HFP, particularly in Norway and Sweden (Supplementary Figure 2). Kendall’s tau between the distance to cities and the frequency a municipality was cited as other place of bite was significant and negative in Norway and Sweden. Supplementary Figure 3 shows the number of respondents by municipality and population density (persons/km^2^).

**Figure 3.**
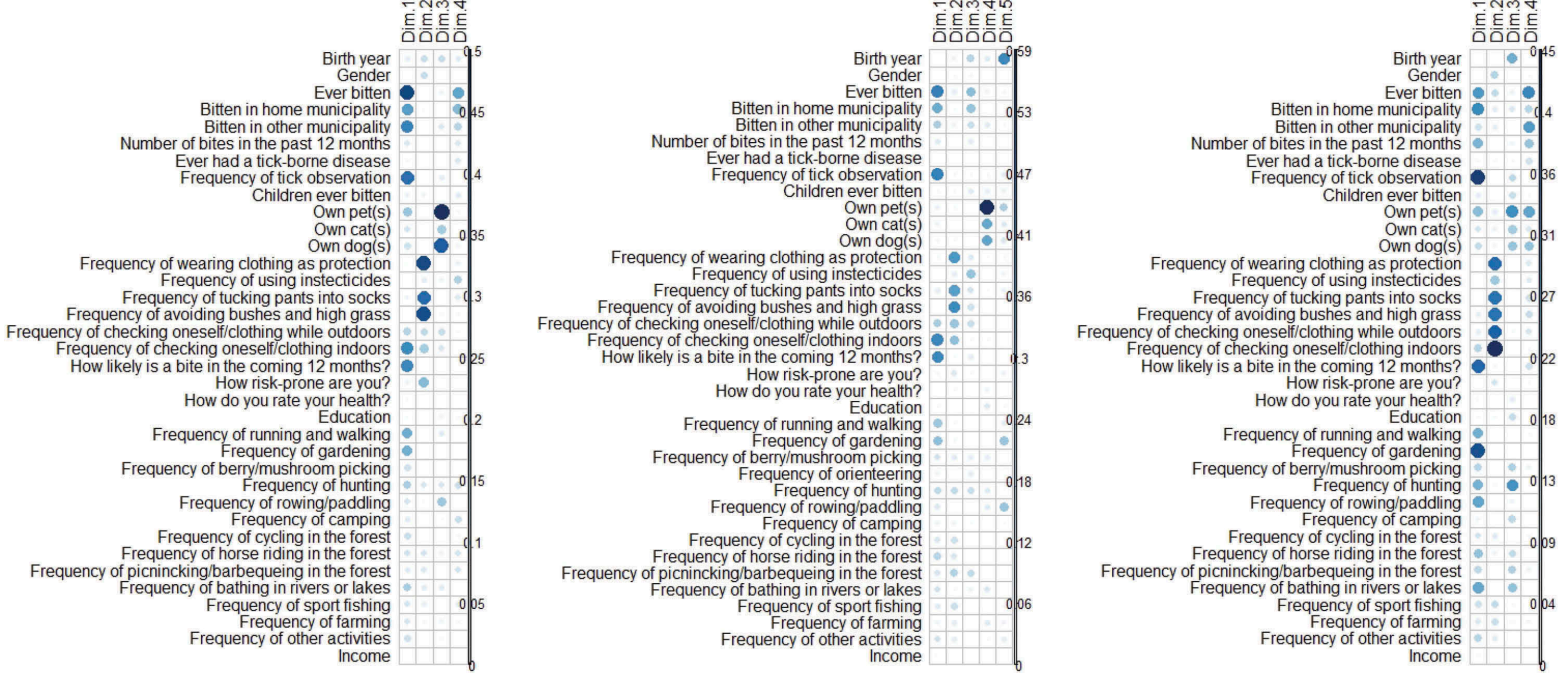
Legend: Correlation indices between original variables and the four (Denmark and Sweden) or five (Norway) dimensions bearing at least 5% of the total variance resulting from the principal component analyses (PCA) at country level on individual responses. Darker blue/larger circle indicate stronger contributions. The figure displays the PCA for Denmark, Norway and Sweden.

### Individual analysis

Neither gender nor age was significantly associated with tick bites in any of the countries, except age-group 18–49 in Denmark, which received more tick bites (Chi^2^, *p* < 0.05). Overall willingness to take risks was associated with tick bites in Norway and Denmark (*p* < 0.05), but not in Sweden. The respondents in Sweden were, in general, less willing to take risks (mean score of 4.6 versus 4.9 in Norway and Denmark) [12]. Self-assessed health status (asked to indicate health status on an ordinal scale) was not associated with tick bites or tick-borne disease (Chi^2^ and PCA; Figure 3). Overall 59% of the respondents had ever been bitten and 60% of those bitten were bitten somewhere else than in their home municipality (Table 4). Eighty-eight percent of the respondents experienced one to five bites over the past 12 months (Table 5). Fifty-three percent of the respondents had children* which had ever been bitten (Table 6), where the great majority had one to five tick bites (Supplementary Table 2). Only 1% of the respondents could not identify the place where they were bitten, and 8% were bitten outside their country of residence (Table 4). Sweden had the highest number of total tick bites and the highest fraction of respondents being bitten (370 bitten out of 1096 respondents/34%) during the last 12 months, followed by Norway (169 bitten out of 789 respondents/21%) and lastly Denmark with 132 bitten out of 783 respondents/17%) (Table 5). Eleven percent reported ever having had a tick-borne disease, and of these, 91% reported LB, 1% TBE and 7% other tick-borne diseases (not specified by all respondents) (Supplementary Table 3). Ninety-five percent (162 cases) of the LB cases were confirmed by diagnostic testing, compared to 50% (1 case) of the TBE cases and 77% (10 cases) of the other tick-borne diseases. Two percent of respondents reported a tick-borne disease during the past 12 months (Supplementary Table 4), of which 82% (23 cases) were LB (96% confirmed by a diagnostic test), 4% (1 case) was TBE (none verified), and 14% (3 cases) were other tick-borne disease (75% verified). The enumeration of tick-borne diseases in children* *If got several children only asked about the oldest child are displayed in Supplementary Tables 5 and 6.

**Table 4.** Number of respondents with tick bites and place of tick bite.

Tick bite	Norway	Denmark	Sweden	Total
Been bitten	389 (49.3%)	407 (52.0%)	781(71.3%)	1577 (59.1%)
Home	199 (51.2%)	192 (47.2%)	479 (60.7%)	870 (55.2%)
Other place	231 (59.4%)	255 (62.7%)	459 (58.8%)	945 (60.0%)
Abroad	25 (6.4%)	49 (12.0%)	45 (5.8%)	119 (7.5%)
Unknown	2 (0.5%)	5 (1.2%)	10 (1.3%)	17 (1.1%)

**Table 5.** Number of tick bites per respondent last 12 months.

Number of tick bites last 12 months	Norway	Denmark	Sweden	Total
1–5	144 (85.2%)	118 (88.7%)	331(89.5%)	593 (88.4%)
6–10	15 (8.9%)	9 (6.8%)	28 (7.6%)	51 (7.6%)
11–15	2 (1.2%)	2 (1.5%)	6 (1.6%)	10 (1.5%)
16–20	1 (0.6%)	1 (0.8%)	3 (0.8%)	5 (0.7%)
Above 20	7 (4.1%)	3 (2.2%)	2 (0.5%)	12 (1.8%)
Total number of respondents	169	133	370	671

**Table 6.** Have you got children under 18 years of age and have they been bitten?

	Norway	Denmark	Sweden	Tot
Got children	258 (33% of respondents)	226 (29% of respondents)	371 (34% of respondents)	855 (32% of respondents)
Bitten*	96 (37%)	122 (54%)	236 (64%)	454 (53%)

*Bitten: if several children the respondent only answers for the oldest one

### Frequency of tick observation and keeping pets

The majority (44%) observed ticks either on themselves or in the environment less than monthly from May-September (Supplementary Table 7). When respondents reported frequency of seeing ticks on their pets the proportion of daily and weekly observation increases (Supplementary Table 8). There is an association between frequency of tick observation and being bitten by a tick (*p* < 0.05 and Dimension 1 of the PCA; Figure 3). The PCA analysis indicated that more frequent tick observation was associated with being bitten in the home municipalities (loadings on Dimension 1 (PCA), Figure 3), and this association was strongest in Sweden. We found no significant association between keeping pets and getting tick bites (Chi^2^) compared to those not having pets.

### Outdoor activities

There is an association (*p* < 0.05 and PCA) between walking/running in the forest several times a week and getting bitten by ticks, compared to those that do not engage in this activity. This association was observed for all three countries. Gardening and paddling/rowing activities were also associated (*p* < 0.05 and PCA) with getting tick bites. Further, picking berries or mushrooms were associated (*p* < 0.05 and PCA) with getting tick bites in Sweden and Denmark, but not Norway. Hunting was also associated (*p* < 0.05 and PCA) with tick bites in Sweden and Denmark, but not Norway. Riding horses was associated (*p* < 0.05 and PCA) with tick bites for Norway and Denmark, but not for Sweden. Going for a picnic, camping, fishing, and swimming were only significant for Denmark (*p* < 0.05 and PCA). Cycling, orienteering, and farming were not associated with getting tick bites (Chi^2^). When looking at tick bite exposure only during the past 12 months, in relation to activity patterns, the same associations exists as with exposure at all times.

### Education and income

Degree of education was associated with getting tick bites in Denmark (*p* < 0.05 and PCA). (increased risk with higher education), but not Norway and Sweden. However, this variable might be confounded by practicing more outdoor activities. In addition, there was no association between income and getting tick bite.

### Protective behavior

Checking the body for ticks both whilst outdoors and afterwards and using socks tucked over trousers were significantly associated with tick bites in all countries (*p* < 0.05). In Denmark, Norway and Sweden, those with a history of bites apply principally body inspection, both indoors and outdoors, and they are concerned about getting future bites (Dimension 1 (PCA) Figure 3). Other protective measures, such as avoidance of bushy areas, were not associated with a history of tick bites (Chi^2^). In Norway, the use of insecticides was associated with a history of getting tick bites (Dimension 3 (PCA), Figure 3). In Sweden, people apply a large selection of protective measures (more than 3 measures), but this was not associated with a history of getting tick bites (PCA).

## Discussion

Published literature on occurrence of tick bites and possible risk factors in the general population is scant. In addition, existing studies have been conducted at small scales and do not give the broad picture of areas of higher risk of tick bites [6–10,18,19]. Our region-wide survey provides a broader view of the issue of tick bites in the human population. This yields useful additional insight into the risk of tick-borne diseases. We found that nearly 60% of adults in Scandinavia reported bites over the previous 12 months (however majority at low levels; one to five bites), a value comparable to previous surveys performed in Sweden and Norway [6–10]. No previous tick bite exposure studies has been published for Denmark. Sweden had the highest fraction of respondents bitten during the last 12 months, compared to Denmark and Norway. The risk of tick bite was hence higher in Sweden compared to Denmark and Norway. Analyses of the protective practices from our questionnaire study [12] showed that respondents from Sweden had two times higher odds to use at least three protective practices compared to the other two countries. They could therefore perhaps be better on protective measures and/or detecting tick bites compared to respondents in Norway and Denmark. However, the fact that Swedish respondents also reported the highest number of tick bites during the last 12 months, highest observed frequencies of ticks both in the environment and on their pets together with the highest numbers of tick-borne diseases and the lowest share of respondents that had never seen a tick, suggest that the actual exposure to ticks and/or tick density might be higher in Sweden. A possible explanation for the difference in tick density and tick bite exposure between Sweden and Norway might be that the population of roe deer is far higher in Sweden, especially in southern Sweden, compared to Norway (Personal communication: Thomas Jaenson and Atle Mysterud) and that roe deer are living in close proximity to inhabited areas; basically in backyards and gardens, so achieve a mixed effect from both increased tick reproduction potential and actual tick exposure. Data from Sweden suggest that the highest tick abundance coincides with the *Limes Norrlandicus ecotone* [4,20], which is in line with findings from our study. Historical baseline levels of tick abundance are however lacking in all three countries, but changes in distribution, which adds to exposure, have been reported from both Sweden and Norway as well as other parts of Europe [21]. In Norway, tick bites are primarily a coastal phenomenon, where population densities (and HFP) are highest, and extending even further up north (Figure 2) than previously assessed in 2009 [3]. The map of Norway is heavily skewed with some municipalities having large number of bites. This is due to a small number of respondents with a large number of bites. Respondents in Sweden reported tick bites homogenously within the distribution range of *Ixodes ricinus*, whilst Denmark had more of an urban/rural contrast. Bites acquired in resident municipality were more numerous in municipalities with a lower human pressure on the environment in all three countries. This was even more pronounced in Denmark, with respondents in municipalities with less human pressure reporting the majority of bites at «home» (Supplementary Figure 1).

From the maps it can be seen that most bites acquired whilst travelling are all in the vicinity of urban centers with high human pressure (Figure 2). This is most visible in Norway and Sweden, with clusters around Oslo, Skien, Kristiansand, Bergen, Stavanger, Stockholm, and Gothenburg. In Denmark, bite frequency was elevated around Copenhagen. This suggests very intense exposure in these areas that might or might not necessarily have high densities of ticks. It is consistent with observations that people tend to travel within the vicinity of their hometown and reflects perhaps primarily strong exposure during recreational activities [22]. It has however been shown that the recreational forest in the suburbs of Copenhagen have high density of ticks [23]. During the recent decades urban transmission of vector-borne disease has increased and there are increasing reports of established tick and pathogen populations in even small remnant forests within urban areas [24]. Urban regions experience unique temperature regimes, termed urban-heat-islands (UHI) [24]. In especially temperate climates UHI can facilitate increased vector development rates [24]. The greatest number of LB cases in the United States occurs often in sub-urban and exurban areas that intersect with forest ecosystems with hosts, vectors, and pathogens [25].

We found two spatial profiles for tick bites: bites acquired in the proximity of the home (summary for respondents bitten in home municipality) and bites acquired while travelling (summary for respondents bitten outside their resident municipality). The first type of location was observed for areas less urbanized/anthropized (as measured by HFP, Supplementary Figure 1), whilst the second type are seen in areas in vicinity of urban centers, where human footprint is higher, tick densities might be lower or higher, but human exposure is however likely to be very high [26] (Supplementary Figure 2). This suggests two distinct spatial profiles: a resident, rural, exposure landscape and visitors to a peri-urban landscape. As our survey was designed for representativity of the overall population, the importance of these spatial profiles in the result is noteworthy. It indicates that urban-dwelling outdoor enthusiasts should as much be the target of prevention campaigns as inhabitants of more rural areas.

The majority of respondents (60% of those bitten) did not get bites in their home municipality, but rather somewhere else within their country. In a survey from Scotland amongst LB patients, where they investigated the location of tick bites [18], only 15% of the patients were bitten at their home address [18]. Thus, disease data on the locations of cases based on residence, rather than location of bite, should be used with caution. A study from the Netherlands show that 43% of people bitten by ticks are bitten in the forest, and 31% are bitten in the garden, without stating if this is in their resident municipality or not [19]. This is similar to our findings regarding running/walking in the forest and gardening being the activities most strongly associated with risk of bites. Additionally, 1 in 5 tick bites in the Netherlands occurred in urban areas [27], which is in line with our findings concerning bites in areas peripheral to the main cities (peri-urban areas), which represent one distinct spatial profile for tick bites in our study.

Running/walking in the forest, gardening, and paddling/rowing were activities most strongly associated with risk of bites. People reporting bites regularly practice a range of outdoors activities, and the weekly frequency of these activities increases with degree of education (results not shown). There is an association between being bitten and running/walking in the woods (activity performed by 80% of respondents), gardening (performed by 70% of respondents), or paddling/rowing (performed by 10% of respondents). Variability among the three countries in the significance of picking mushroom/berries (around 30%), hunting (around 5%), and riding (around 2%) as risk factors for tick bites is challenging to explain. Hunting and picking mushrooms are however late autumn activities, when lower tick abundance is expected, and also people tend to use long trouser/long sleeves at this time of year. Neither cycling (around 40%), orienteering (around 4%), nor farming (around 1%) activities were associated with bites, and swimming (around 55%) was only significant for Denmark. It is likely that the less common activities had insufficient sample size for a consistent or powerful effect. Protective measures, such as checking body for ticks (indoor/outdoor) and tucking pants into socks, were associated with bites, this might be due to increased uptake of such measures in areas of high tick density and by people with a history of tick bites who thus protect themselves.

The significance of frequency of tick observation in the environment and being bitten by a tick, implies that frequency of observation of ticks in an area might be used as a proxy for the actual risk of exposure to tick bites. This association could give leads for awareness-raising by encouraging people to be vigilant if ticks observed on pets or in the environment. Most respondents reported seeing ticks on themselves or in the environment ‘less than monthly’, whilst most observed ticks on their pets ‘weekly’, and hence pets might seem to be an even more sensitive indicator of human risk. It is well established that pets can act as more sensitive sentinel indicators for both tick bites and disease risk for humans [28–30]. It has additionally been suggested that spatial differences in tick density can be estimated by the number of ticks per pet [20]. We know that across Europe *Ixodes ricinus* typically make up 90–100% of all ticks removed from humans [8,31–33]. In addition, collected ticks from dogs and cats in Norway showed that 99% was *Ixodes ricinus* [34], similar percentage was found also in ticks collected in Sweden [20,35] and Denmark [36]. Studies from Great Britain and Belgium reports 89% and 76% respectively and dogs restricted to urban habitats were no less likely to have ticks than dogs from rural habitats [37,38].

Finally, our analysis shows that the first five components of the PCA bear only about 20% of the data variability, which indicates that many more variables are needed to unravel the complex nature of tick bite associations. In assessing risk activities, the less common activities likely had insufficient sample size for a consistent or powerful effect. Other shortcomings are that the respondents might not be completely representative of the general population, with slightly more women, a more well-educated population, and a self-selected population with particular interest in the topic than average, in addition to nil/low number of respondents for certain municipalities. A low number of respondents per municipality mean that municipality-level indicators may be heavily affected by extreme observations, as is visible on the map of Norway with some municipalities having large number of bites, in relation to a small number of respondents with a large number of bites. As for all such studies, there is also recall bias and, being a cross-sectional survey, there are no temporal details, so it is impossible to determine causality of a certain behavior linked to tick bite.

Until now, two main sources of data have been used to study the risk of tick-borne diseases: prevalence of pathogens in ticks, and disease data from public health registries. Prevalence data from ticks are usually of limited spatial and temporal coverage and usually lack representativity if sampled by dragging (around 6% efficiency [39] and primarily catching nymphs). Therefore, since efficiency is so low, it cannot be used to verify presence or absence. Disease data, on the contrary, are spatially exhaustive, but factors as location by residence uncertainty, delays, and the fact that only symptomatic infections are displayed must be taken into account. In addition any such data have rather poor representation of human-tick contacts. It is no trivial matter to understand the intensity of human exposure, since researchers often are limited to small-scale studies of tick-human-biting exposure. Avoiding spatial and temporal bias is challenging for citizen-collected data. Thus, numerous different data collection efforts, including tick-exposure and associated risk-activities, are needed to reconcile information on local disease risk. Our findings should be considered when developing prevention strategies to reduce both tick bites and tick-borne disease cases, for example; targeting information campaigns to the general public differently for residents and visitors.

## Conclusion

We found that tick bites affect the entire Scandinavian population, with higher risk of tick bites in Sweden compared to Denmark and Norway. Our results show that nearly 60% of adults reported tick bites, and that the majority were not bitten in their resident municipality. This implies that disease data on the locations of cases based on residence should be used with caution. In home municipalities, people were most often bitten in less, but not the least, urbanized areas. When visiting other municipalities, people were most frequently bitten in peri-urban areas, likely due to their attraction for recreating urban dwellers. Running/walking in the forest, gardening, and paddling/rowing were the activities most strongly associated with risk of bites. Only 1% of the respondents could not identify the place where they think the tick bite(s) occurred. Our results suggest targeting regular practitioners of specific outdoor activities for preventive messaging and that we also need to provide different kind of information to people regarding location of exposure at home versus when visiting peri-urban areas. The findings also imply that observations of ticks, are linked to likelihood of tick bites, and that observations of ticks on pets, versus people or in the environment, may be an even more sensitive indicator of human risk.

## Supplementary Material

Supplemental Material

## Data Availability

The data are accessible upon request.
