# Supplementary material for: Spatial tick bite exposure and associated risk factors in Scandinavia
Source: Infect Ecol Epidemiol. 2020 Jun 7;10(1):1764693. doi: 10.1080/20008686.2020.1764693 (PMC7448850; doi:10.1080/20008686.2020.1764693)

**Supplementary Figure 1. Sweden: Mean number of bites acquired in the home municipality, by municipality and mean Human Footprint, by municipality**


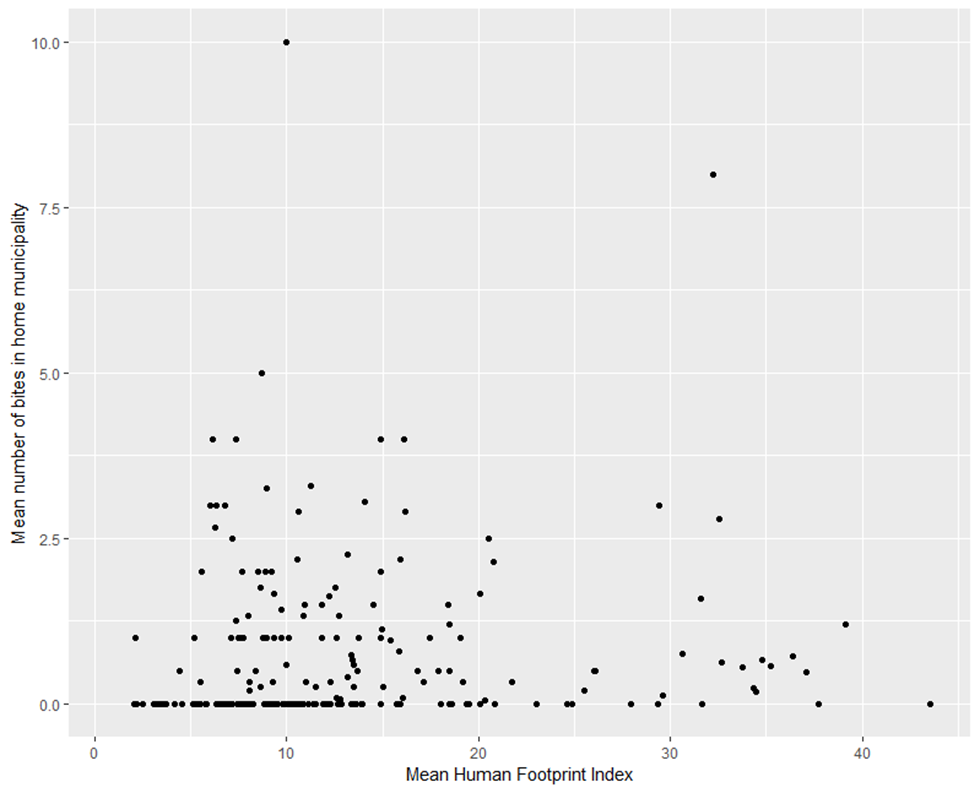

Supplement: Supplemental Material [file ZIEE_A_1764693_SM5029.zip › Supplementary/Supplementary/Supplementary_Figure_1_SE.docx]
