# Supplementary material for: Spatial tick bite exposure and associated risk factors in Scandinavia
Source: Infect Ecol Epidemiol. 2020 Jun 7;10(1):1764693. doi: 10.1080/20008686.2020.1764693 (PMC7448850; doi:10.1080/20008686.2020.1764693)
Supplement: Supplemental Material [file ZIEE_A_1764693_SM5029.zip › Supplementary/Supplementary/Supplementary_Figure_2_SE.docx]

**Supplementary Figure 2. Sweden: Number of times a municipality was cited as place of bite other than residence, by distance to a main town and mean Human Fooprint by municipality**

**
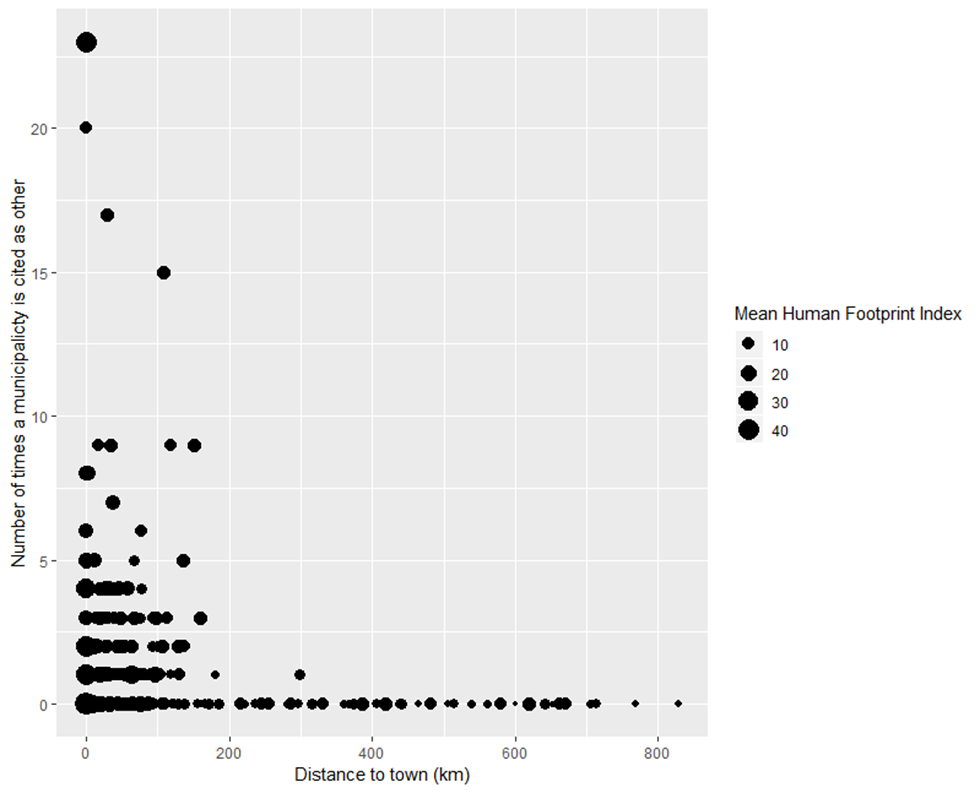
**
