# Supplementary material for: Spatial tick bite exposure and associated risk factors in Scandinavia
Source: Infect Ecol Epidemiol. 2020 Jun 7;10(1):1764693. doi: 10.1080/20008686.2020.1764693 (PMC7448850; doi:10.1080/20008686.2020.1764693)
Supplement: Supplemental Material [file ZIEE_A_1764693_SM5029.zip › Supplementary/Supplementary/Supplementary_Table_1.docx]

| Kendall’s tau (p-value) | Distance to town (km)*Frequency a municipality is mentioned as other place of bite | Mean Human Footprint Index* Frequency a municipality is mentioned as other place of bite | Mean Human Footprint Index*Mean number of bites home |
| --- | --- | --- | --- |
| Denmark | -0.11 (0.15) | 0.10 (0.17) | -0.13 (0.07) |
| Norway | **-0.16(2.08e-05)** | **0.27(<0.01)** | **0.13 (0.01)** |
| Sweden | **-0.25 (<0.01)** | -0.06(0.13) | **0.12 (0.02)** |

**Supplementary Table 1**
