# Supplementary material for: Spatial tick bite exposure and associated risk factors in Scandinavia
Source: Infect Ecol Epidemiol. 2020 Jun 7;10(1):1764693. doi: 10.1080/20008686.2020.1764693 (PMC7448850; doi:10.1080/20008686.2020.1764693)
Supplement: Supplemental Material [file ZIEE_A_1764693_SM5029.zip › Supplementary/Supplementary/Supplementary_Table_2.docx]

**Supplementary Table 2: How many tick bites have your child*had during the last 12 months?**

| **Children* under 18 years old** | **Norway** | **Denmark** | **Sweden** | **Total** |
| --- | --- | --- | --- | --- |
| 1-5 tick bites | 51 | 63 | 139 | 253 |
| 6-10 tick bites | 4 | 6 | 11 | 21 |
| 11-15 tick bites | 1 | 0 | 1 | 2 |
| 16-20 tick bites | 0 | 1 | 0 | 1 |
| Above 20 tick bites | 1 | 0 | 0 | 1 |

* If several children, only answer for the oldest child
