# Supplementary material for: Spatial tick bite exposure and associated risk factors in Scandinavia
Source: Infect Ecol Epidemiol. 2020 Jun 7;10(1):1764693. doi: 10.1080/20008686.2020.1764693 (PMC7448850; doi:10.1080/20008686.2020.1764693)
Supplement: Supplemental Material [file ZIEE_A_1764693_SM5029.zip › Supplementary/Supplementary/Supplementary_Table_3.docx]

**Supplementary Table 3: Have you ever had tick-borne disease?**

| **Adults** | **Norway** | **Denmark** | **Sweden** | **Total** |
| --- | --- | --- | --- | --- |
| Number of respondents | 36 | 30 | 119 | 185 |
| Lyme borreliosis | 27 (26*) | 26 (24*) | 115 (109*) | 168 |
| TBE | 0 | 0 | 2 (1*) | 2 |
| Other tick-borne disease | 6 (5*) | 4* | 3 (1*) | 13 |
| Do not know | 3 | 0 | 2 | 5 |

*****Verified by General practitioner/lab
