# Supplementary material for: Spatial tick bite exposure and associated risk factors in Scandinavia
Source: Infect Ecol Epidemiol. 2020 Jun 7;10(1):1764693. doi: 10.1080/20008686.2020.1764693 (PMC7448850; doi:10.1080/20008686.2020.1764693)
Supplement: Supplemental Material [file ZIEE_A_1764693_SM5029.zip › Supplementary/Supplementary/Supplementary_Table_4.docx]

**Supplementary Table 4: Have you had tick-borne disease during the last 12 months?**

| **Adults** | **Norway** | **Denmark** | **Sweden** | **Total** |
| --- | --- | --- | --- | --- |
| Number of respondents | 6 | 5 | 17 | 28 |
| Lyme borreliosis | 3* | 4* | 16 (15*) | 23 |
| TBE | 0 | 0 | 1 | 1 |
| Other tick-borne disease | 3* | 0 | 1 | 4 |

*****Verified by General practitioner/lab
