# Supplementary material for: Spatial tick bite exposure and associated risk factors in Scandinavia
Source: Infect Ecol Epidemiol. 2020 Jun 7;10(1):1764693. doi: 10.1080/20008686.2020.1764693 (PMC7448850; doi:10.1080/20008686.2020.1764693)
Supplement: Supplemental Material [file ZIEE_A_1764693_SM5029.zip › Supplementary/Supplementary/Supplementary_Table_5.docx]

**Supplementary Table 5: Have your child ever had tick-borne disease?**

| **Children under 18 years old** | **Norway** | **Denmark** | **Sweden** | **Total** |
| --- | --- | --- | --- | --- |
| Total number | 6 | 1 | 14 | 21 |
| Lyme borreliosis | 4 (2*) | 1* | 13* | 18 |
| TBE | 0 | 0 | 0 | 0 |
| Other tick-borne disease | 2* | 0 | 1* | 3 |

(If got several children only report for the oldest child)

*Verified by GP/lab
