# Supplementary material for: Spatial tick bite exposure and associated risk factors in Scandinavia
Source: Infect Ecol Epidemiol. 2020 Jun 7;10(1):1764693. doi: 10.1080/20008686.2020.1764693 (PMC7448850; doi:10.1080/20008686.2020.1764693)
Supplement: Supplemental Material [file ZIEE_A_1764693_SM5029.zip › Supplementary/Supplementary/Supplementary_Table_6.docx]

**Supplementary Table 6: Have your child had tick-borne disease during the last 12 months?**

| **Children under 18 years old** | **Norway** | **Denmark** | **Sweden** | **Total** |
| --- | --- | --- | --- | --- |
| Total number | 1 | 2 | 2 | 5 |
| Lyme borreliosis | 0 | 1* | 2* | 3 |
| TBE | 0 | 0 | 0 | 0 |
| Other tick-borne disease | 0 | 1* | 0 | 1 |
| Do not know | 1 | 0 | 0 | 1 |

*****Verified by General Practitioner/lab

(If got several children only report for the oldest child)
