# Supplementary material for: Spatial tick bite exposure and associated risk factors in Scandinavia
Source: Infect Ecol Epidemiol. 2020 Jun 7;10(1):1764693. doi: 10.1080/20008686.2020.1764693 (PMC7448850; doi:10.1080/20008686.2020.1764693)
Supplement: Supplemental Material [file ZIEE_A_1764693_SM5029.zip › Supplementary/Supplementary/Supplementary_Table_7.docx]

**Supplementary Table 7: Frequency of tick observation from May to September**

| **Frequency** | **Norway** | **Denmark** | **Sweden** | **Total** |
| --- | --- | --- | --- | --- |
| Daily | 16 (3%) | 36 (6%) | 63 (6%) | 115 (4%) |
| Weekly | 103 (17%) | 106 (17%) | 224 (23%) | 433 (16%) |
| Monthly | 147 (24%) | 123 (20%) | 234 (24%) | 504 (19%) |
| Less then monthly | 335 (56%) | 363 (58%) | 464 (47%) | 1162 (44%) |
| Never seen | 150 (19%) | 107 (14%) | 68 (6%) | 325 (12%) |
| Do not know | 38 (5%) | 48 (6%) | 43 (4%) | 129 (5%) |
| Total number of respondents | 789 | 783 | 1096 | 2668 |
