# Supplementary material for: Spatial tick bite exposure and associated risk factors in Scandinavia
Source: Infect Ecol Epidemiol. 2020 Jun 7;10(1):1764693. doi: 10.1080/20008686.2020.1764693 (PMC7448850; doi:10.1080/20008686.2020.1764693)
Supplement: Supplemental Material [file ZIEE_A_1764693_SM5029.zip › Supplementary/Supplementary/Supplementary_Table_8.docx]

**Supplementary Table 8: Frequency of observation of ticks on outdoor pets**

| **Frequency** | **Norway** | **Denmark** | **Sweden** | **Total** |
| --- | --- | --- | --- | --- |
| Daily | 17 (6%) | 31 (10%) | 61 (16%) | 109 (11%) |
| Weekly | 72 (25%) | 77 (26%) | 151 (39%) | 300 (31%) |
| Monthly | 59 (20%) | 70 (23%) | 78 (20%) | 207 (21%) |
| Less then monthly | 77 (27%) | 84 (28%) | 66 (17%) | 227 (23%) |
| Never seen | 63 (22%) | 36 (12%) | 27 (7%) | 126 (13%) |
| Total number of respondents | 288 | 298 | 383 | 969 |
